# Supplementary material for: Determinants of the food insecurity at household level in Pakistan: A multilevel model approach
Source: PLoS One. 2023 Oct 5;18(10):e0291343. doi: 10.1371/journal.pone.0291343 (PMC10553256; doi:10.1371/journal.pone.0291343)
Supplement: S1 Data — (DOCX) [file pone.0291343.s001.docx]

**Appendix A: Food Insecurity Experience Scale – Survey Module**

| **Item No.** | **Item Questions/description** | **Item abbreviation** | **Domain** | **Mean value** |
| --- | --- | --- | --- | --- |
| 1 | During the last 12 months, was your household worried any time that your food would run out before you had money to buy more? 1 = yes 0 = No | Worried | Anxiety, insecurity | 0.596 |
| 2 | During the last 12 months, the food your household had did not last, and you did not have enough money to buy more? 1 = yes 0 = No | Did not last/ran out | Anxiety, insecurity | 0.552 |
| 3 | During the last 12 months, did your or any other adult in your household cut the size of your meals because you did not have enough money to buy food? 1 = yes 0 = No | Cut size | Quantity | 0.452 |
| 4 | During the last 12 months, did you or other member of your household skip some of daily meals because you did not have enough money for food? 1 = yes 0 = No | Skip | Quantity | 0.216 |
| 5 | During the last 12 months, did you ever eat less than you felt you should because you did not have enough money to buy food? 1 = yes 0 = No | Eat less | Quantity | 0.310 |
| 6 | During the last 12 months, were you or other of your household ever hungry and did not eat because you did not have money to buy enough food? 1 = yes 0 = No | Hungry | Consequence of reduced intake | 0.163 |
| 7 | During the last 12 months, did you lose weight because you did not have enough money to buy food? 1 = yes 0 = No | Lose weight | Consequence of reduced intake | 0.237 |
| 8 | During the last 12 months, did you or another adult in your household ever not eat for a whole day because you did not have enough money to buy food? 1 = yes 0 = No | Whole day | Quantity | 0.128 |

**Appendix B: Definition of variables**

| **Variables** | **Definition of variables** |
| --- | --- |
| **Outcome variables** | |
| Four categories: | Category 1 = Food Secure Household (raw score, 0);  Category 2 = Mild Food Insecure Household (raw score, 1-3);  Category 3 = Moderate Food Insecure Household (raw score, 4-6);  Category 4 = Severe Food Insecure Household (7-9) |
| Food Insecure Household | Moderate Food Insecure Household Plus Severe Food Insecure Household = 1, zero otherwise |
| Severe Food Insecure Household | Severe Food Insecure Household = 1, zero otherwise |
| **Explanatory variables** | |
| Gender | Gender of the household; female =1, male = 0 |
| Age | Household head age and age-squared |
| Education | Years of schooling (from no education to highest level) |
| Household size | Household size is used as a continuous variable |
| Log of per capita consumption | Household natural log of per capita consumption used as proxy for household per capita income per month |
| Employment level | We categorize employment status of the household head into whether household head is employed, farmer or run his own business.  Yes = 1, No = 0 |
| Own residence | Whether household has its own house?  Yes = 1, No = 0 |
| Agriculture land | Did the father of the head of household owned agriculture land? Yes = 1, No = 0 |
| Shocks | Has your household affected during the last five years from any shocks such as drought, flood, earthquake, inflation etc.? Yes = 1, No = 0 |
| Inflation shock | How severely has your household been affected by the recent food price increase/high overall inflation? Five categories; 1 = not at all affected, 2 = mildly affected, 3 = moderately affected, 4 = highly affected, 5 = severely affected |
| Experience casualities | Did any member in your village get any injury during the last 12 months?  Yes =1, No =0 |
| Region | We have two region, Urban = 1, Rural = 0 |
| Provinces | Province-1 = Punjab, province-2 = Sindh, province-3 = KP, province-4 = Baluchistan |

**Appendix C: Determinants of food insecurity (multi-level null and random slope models)**

| **Variables** | **Model-1^a^** | **Model-2^b^** | **Mdoel-3^c^** | **Model-4^d^** |
| --- | --- | --- | --- | --- |
| Gender of the household head (Reference Category male) | | |  |  |
| Female |  | 0.076** (0.033) |  | 0.056** (0.026) |
| Age of the household head as continuous variable | | | | |
| Age |  | -0.005** (0.002) |  | -0.002*** (0.002) |
| Age Square |  | 0.000 (0.000) |  | 0.000 (0.000) |
| Household size as continuous variable | | | | |
| Household size |  | -0.014*** (0.001) |  | -0.009*** (0.001) |
| Education as continuous variable | | |  |  |
| Education |  | -0.001*** (0.001) |  | -0.006*** (0.001) |
| Household income as continuous variables | | | | |
| Income |  | -0.102*** (0.011) |  | -0.064*** (0.018) |
| Experience shocks during the last five years (Reference Category No) | | | | |
| Yes |  | 0.083*** (0.023) |  | -0.013 (0.015) |
| Experience (Reference Category No inflation) | | | | |
| Mild inflation |  | -0.009 (0.028) |  | -0.013 (0.022) |
| Moderate inflation |  | -0.077* (0.045) |  | -0.063* (0.036) |
| Hihgly inflation |  | 0.228*** (0.028) |  | 0.081*** (0.022) |
| Severe inflation |  | 0.182*** (0.041) |  | 0.167*** (0.033) |
| Experience injury in the village (Reference Category No) | | | | |
| Yes |  | 0.005 (0.019) |  | 0.013 (0.015) |
| Constant |  | 1.293*** (0.123) |  | 0.773*** (0.097) |
| **Variance at district, community, household and casualty at community level** | | | | |
| SD (district) | 0.199*** (0.038) | 0.177** 0.0.034) | 0.146*** (0.027) | 0.126*** (0.023) |
| SD (community) | 0.161*** (0.011) | 0.125 *** (0.010) | 0.090*** (0.008) | 0.072*** (0.008) |
| SD (household) | 0.433***(0.004) | 0.401*** (0.004) | 0.335*** (0.003) | 0.324*** (0.003) |
| SD (Community injury) |  | 0.108*** (0.024) |  | 0.091*** (0.018) |
| **Diagnostic test** | | | | |
| Wald chi2 (17) |  | 667.92 (12) |  | 259.93 (12) |
| Prob > chi2 | 0.000 | 0.000 |  |  |
| LR test vs. probit model: chi2 (3) | 759.32*** | 582.54*** | 706.36*** | 491.53*** |

1. Null model for *Food Insecure Household* estimation
2. *Food Insecure Household* estimation
3. Null Model for *Severe Food Insecure Household* estimation
4. *Severe Food Insecure Household* estimation
