# Supplementary material for: Determinants of the food insecurity at household level in Pakistan: A multilevel model approach
Source: PLoS One. 2023 Oct 5;18(10):e0291343. doi: 10.1371/journal.pone.0291343 (PMC10553256; doi:10.1371/journal.pone.0291343)
Supplement: Attachment — Submitted filename: Plos One.pdf [file pone.0291343.s004.pdf]

#Minor correction

Overview: This manuscript discusses the determinants of the food insecurity at household level in Pakistan through multilevel model approach. Research methods include a questionnaire survey, and variance analysis. Overall, there are several issues with the manuscript, and I suggest that the authors thoroughly revise the manuscript based on the following comments.

Specific comments

- ✓ Its better if abstract contains some significant statistical result/value in result sections.
- ✓ Currently, the research background and methods have not been introduced in detail. The results of the study need to be condensed and are somewhat lengthy, and re-write the conclusion section in abstract.
- ✓ The Research conducted year needed in the abstract and methodology section.

- It is better use other representative word in key word section, that is different from the *title of article*.
- It is better use the word “Introduction” in place of “Background”
- Page 3, line 37 presented as “Ending hunger and malnutrition is the 38 most daunting global problem as 828 million populations are facing food insecurity in 2022 [11]”, is it the global or national? If it global you needed to put the national also.
- Page 4; line 74 , use the word “Methods and materials” instead of “DATA AND EMPIRICAL METHOD”
- In this section you need to put the description of selected study area in mean of both map and its representation. It is better if in this way “***Study design, period and study area, Total population, inclusion and exclusion criteria for the selected sample respondent; Sample size determination and sampling procedure; Data collection, quality and management and Data analysis*** ”

1. Differentiate the result from discussion, make discussion other newu section, the discuss the significant finding only, minimize the redundancies.
2. Page 8, line 175 “(Table 1) presents the….” Re-write it.
3. Page 8 line 195 “(Table 2) presents the results of the multilevel mixed-effects generalized linear models for 196 both food-insecure households and severe food-insecure households….” Rewrite it.
4. In this result and discussion section you used the unnecessary Italic paragraphs and words. Consider it again.
5.

   3\. Experiments, statistics, and other analyses are performed to a high technical standard and are described in sufficient detail.

6. Put Limitation of the study and future research directions
7. Spilt “CONCLUSION AND POLICY IMPLICATION” section into “Conclusion” and. “***Implications of the study for practice and/or policy”***
8. *You highly encourage to discuss your findings with the following relevant works:*

   http://dx.doi.org/10.1016/j.jafr.2023.100597

   http://dx.doi.org/10.1016/j.jafr.2023.100500

   http://dx.doi.org/10.1016/j.jafr.2022.100461

   http://dx.doi.org/10.1016/j.jafr.2023.100610
9. You may shift Appendix to supplementary data source.

This all

Good Lack
