# Supplementary material for: Determinants of the food insecurity at household level in Pakistan: A multilevel model approach
Source: PLoS One. 2023 Oct 5;18(10):e0291343. doi: 10.1371/journal.pone.0291343 (PMC10553256; doi:10.1371/journal.pone.0291343)
Supplement: Attachment — Submitted filename: Nkoko PLOS Manuscript comments.docx [file pone.0291343.s005.docx]

Dear Editor

Thank you for the opportunity to review the manuscript - Determinants of the Food Insecurity at Household level in Pakistan: A Multilevel Model Approach.

**General comments:**

- The paper is well written, even though the authors have used old data, the food insecurity situation may have changed a lot to this point. Yet I imagine it is beneficial to understand the historical food insecurity trends to inform interventions and policy making.
- Language editing is recommended as some areas are bit difficult to read.
- I recommend the manuscript for publication, subsequent to attending to minor corrections

**Abstract**

Line 5 – are the determinants and risks for food insecurity at a national or household level?

Please rephrase line 13-14 it reads a bit difficult

**Introduction**

Please elaborate more on the food insecurity situation in Pakistan using recent sources

Add citation for line 63 to 66

Please present the background a bit more orderly – the points are a bit haphazard, maybe paragraphs can each address a theme e.g. the nature of food insecurity, SGDs and food insecurity, measurement of food insecurity, global and national food insecurity trends etc.

There is repetition on line 29-30 and 63-64

Kindly show who will benefit from this work

**Methodology**

Line 91 – kindly clarify, is it the head of the household who asked or responded to the questions?

Line 203 – less/fess utilization?

The stated percentages in lines 175-190 are not evident in Table 1

**Results and discussion**

Please discuss further using recent studies and clearly show comparisons of food insecurity and determinants in other countries

**Conclusions**

Kindly include the prevalence of food insecurity in the conclusion, as it has been stated in the background that the prevalence will be assessed
