# Supplementary material for: Determinants of the food insecurity at household level in Pakistan: A multilevel model approach
Source: PLoS One. 2023 Oct 5;18(10):e0291343. doi: 10.1371/journal.pone.0291343 (PMC10553256; doi:10.1371/journal.pone.0291343)
Supplement: Attachment — Submitted filename: Response to reviwers.docx [file pone.0291343.s006.docx]

Manuscript Title: **Determinants of the Food Insecurity at Household level in Pakistan: A Multilevel Model Approach**

Authors are grateful to the reviewers for their enlightening comments on our previous manuscript submitted to PloS One. We have thoroughly revised the manuscript in light of the comments of the reviewers. Reviewer’s comments and author’s response is provided below;

| **#** | **Comments** | **Response** | **Page #** |
| --- | --- | --- | --- |
| **Journal requirement** | | | |
| 1 | Please ensure that your manuscript meets PLOS ONE's style requirements, including those for file naming. The PLOS ONE style templates can be found at | Corrected as per the provided style | Whole paper |
| 2 | "We Acknowledge the support from Rachadapisek Sompote Fund for Postdoctoral Fellowship, Chulalongkorn University Thailand, and support for doctoral research university of Gottingen Germany"  We note that you have provided funding information that is not currently declared in your Funding Statement. However, funding information should not appear in the Acknowledgments section or other areas of your manuscript. We will only publish funding information present in the Funding Statement section of the online submission form.  Please remove any funding-related text from the manuscript and let us know how you would like to update your Funding Statement. Currently, your Funding Statement reads as follows:  "The author(s) received no specific funding for this work."  Please include your amended statements within your cover letter; we will change the online submission form on your behalf. | Thank you very much for providing this guidance. We declare that this research was not funded but was the part of our doctoral research project. That’s why we have included acknowledgement for our doctoral and postdoc support program. Kindly allow us to include this as acknowledgement in our paper. Moreover, we are very clear that author received no funding for this work. | 14 |
| 3 | In your Data Availability statement, you have not specified where the minimal data set underlying the results described in your manuscript can be found. PLOS defines a study's minimal data set as the underlying data used to reach the conclusions drawn in the manuscript and any additional data required to replicate the reported study findings in their entirety. All PLOS journals require that the minimal data set be made fully available. | We have added this in the paper. The datasets used is publicly available. <http://www.pidelms.com/pphs/applyfordata.php> | 14 |
| 4 | Your ethics statement should only appear in the Methods section of your manuscript. If your ethics statement is written in any section besides the Methods, please delete it from any other section. | Removed and added in method section | 5 |
| 5 | Please ensure that you refer to Figure 1 in your text as, if accepted, production will need this reference to link the reader to the figure. | Figure is included in the manuscript. | 7 |
| 6 | Please review your reference list to ensure that it is complete and correct. If you have cited papers that have been retracted, please include the rationale for doing so in the manuscript text, or remove these references and replace them with relevant current references. Any changes to the reference list should be mentioned in the rebuttal letter that accompanies your revised manuscript. If you need to cite a retracted article, indicate the article’s retracted status in the References list and also include a citation and full reference for the retraction notice. | Checked and edited as per the reviewer’s comments. |  |
| **Reviewer 1** | | | |
|  | This manuscript discusses the determinants of the food insecurity at household level in Pakistan through multilevel model approach. Research methods include a questionnaire survey, and variance analysis. Overall, there are several issues with the manuscript, and I suggest that the authors thoroughly revise the manuscript based on the following comments. |  |  |
|  | **Specific comments**  Its better if abstract contains some significant statistical result/value in result sections. | Many thanks; we have revised as per the suggestions. | 2 |
|  | Currently, the research background and methods have not been introduced in detail. The results of the study need to be condensed and are somewhat lengthy, and re-write the conclusion section in abstract. | Revised abstract for better understanding. | 2 |
|  | The Research conducted year needed in the abstract and methodology section. | This study was conducted in 2023, added in abstract and methodology section. | 2, 4 |
|  | It is better use other representative word in key word section that is different from the title of article. | Revised and added representative words | 2 |
|  | It is better use the word “Introduction” in place of “Background” | Corrected as per guidance. | 3 |
|  | Page 3, line 37-38 presented as “Ending hunger and malnutrition is the most daunting global problem as 828 million populations are facing food insecurity in 2022 [11]”, is it the global or national? If it global you needed to put the national also. | We have added following statement in our manuscript for national issue.  “Around 16% of the population is facing the problem of moderate and severe food insecurity in Pakistan” | 3 |
|  | Page 4; line 74 , use the word “Methods and materials” instead of “DATA AND EMPIRICAL METHOD” | Corrected | 4 |
|  | In this section you need to put the description of selected study area in mean of both map and its representation. It is better if in this way “Study design, period and study area, Total population, inclusion and exclusion criteria for the selected sample respondent; Sample size determination and sampling procedure; Data collection, quality and management and Data analysis ” | Thanks for the guidance, we have followed this instruction and edited our paper accordingly. | 4-8 |
| 1 | Differentiate the result from discussion, make discussion other new section, the discuss the significant finding only, minimize the redundancies. | We have separated results and discussion section as per the suggestion. | 11-13 |
| 2 | Page 8, line 175 “(Table 1) presents the….” Re-write it | Corrected | 8 |
| 3 | Page 8 line 195 “(Table 2) presents the results of the multilevel mixed-effects generalized linear models for 196 both food-insecure households and severe food-insecure households….” Rewrite it. | Corrected | 10 |
| 4 | In this result and discussion section you used the unnecessary Italic paragraphs and words. Consider it again. | Edited and corrected as per the suggestions | |
| 5 | Experiments, statistics, and other analyses are performed to a high technical standard and are described in sufficient detail. | Many thanks for appreciation our work. | |
| 6 | Put Limitation of the study and future research directions | Added | 13 |
| 7 | Spilt “CONCLUSION AND POLICY IMPLICATION” section into “Conclusion” and. “Implications of the study for practice and/or policy” | We have split in two sections as per the guidance. | 13 |
| 8 | You highly encourage to discuss your findings with the following relevant works:  http://dx.doi.org/10.1016/j.jafr.2023.100597 http://dx.doi.org/10.1016/j.jafr.2023.100500 http://dx.doi.org/10.1016/j.jafr.2022.100461 http://dx.doi.org/10.1016/j.jafr.2023.100610 | Thanks for identifying these relevant literature, we have incorporated in our discussion part. | 11-13 |
| 9 | You may shift Appendix to supplementary data source. | Shifted as supplementary data |  |
| **Reviewer 2** | | | |
| 3 | Thank you for the opportunity to review the manuscript - Determinants of the Food Insecurity at Household level in Pakistan: A Multilevel Model Approach.  General comments:  •The paper is well written, even though the authors have used old data, the food insecurity situation and determinants may have changed a lot to this point. Yet I imagine it is beneficial to understand the historical food insecurity trends to inform interventions and policy making. | Many thanks for appreciation and consideration of our important research work that will be beneficial for the policy makers in the future in Pakistan. | |
|  | Language editing is recommended as some areas are bit difficult to read. | Language edited by the native speaker and corrected accordingly. | Whole paper |
|  | I recommend the manuscript for publication, subsequent to attending to minor corrections  Abstract  Line 5 – are the determinants and risks for food insecurity at a national or household level?  Please rephrase line 13-14 it reads a bit difficult | Many thanks; we have revised the manuscript as per the comments received by the valuable reviewers.  Word household level is added for more clarity.  Revised.  2 | |
|  | Introduction Please elaborate more on the food insecurity situation in Pakistan using recent sources  Add citation for line 63 to 66 | Added | 4 |
|  | Please present the background a bit more orderly – the points are a bit haphazard, maybe paragraphs can each address a theme e.g. the nature of food insecurity, SGDs and food insecurity, measurement of food insecurity, global and national food insecurity trends etc.  There is repetition on line 29-30 and 63-64 | Revised as per the suggestion.  Many thanks for identifying this mistake, we have deleted this repeated sentence. | 3 |
|  | Kindly show who will benefit from this work | Added in introduction and conclusion that the policy makers, farmers and landlord will use this evidence for to address this food insecurity issue in Pakistan. | 4,13 |
|  | Methodology Line 91 – kindly clarify, is it the head of the household who asked or responded to the questions?  Line 203 – less/fess utilization? | Yes, head od household were the respondent in this study.  Thanks. This was typo error, Corrected as “less”. | 5  10 |
|  | The stated percentages in lines 175-190 are not evident in Table 1 | We have included this roundup figure in the table-1 for more clarity. | 8,9 |
|  | Results and discussion  Please discuss further using recent studies and clearly show comparisons of food insecurity and determinants in other countries | We have separated results and discussion section and added more literature for clarity purpose. | 11-13 |
|  | Conclusions Kindly include the prevalence of food insecurity in the conclusion, as it has been stated in the background that the prevalence will be assessed | Thanks. We have revised and added prevalence as per the suggestions. | 13 |
