# Supplementary material for: Determinants of the food insecurity at household level in Pakistan: A multilevel model approach
Source: PLoS One. 2023 Oct 5;18(10):e0291343. doi: 10.1371/journal.pone.0291343 (PMC10553256; doi:10.1371/journal.pone.0291343)
Supplement: Attachment — Submitted filename: Reco-plos One.docx [file pone.0291343.s007.docx]

Dear editor in chief,

Thank you for your consideration to review this relevant work!

The author made a potential modification in the revised version and now in the status is good for publication.

Dawit Leja

**Regards!**
