# Supplementary material for: Determinants of the food insecurity at household level in Pakistan: A multilevel model approach
Source: PLoS One. 2023 Oct 5;18(10):e0291343. doi: 10.1371/journal.pone.0291343 (PMC10553256; doi:10.1371/journal.pone.0291343)
Supplement: Attachment — Submitted filename: Response to reviwers.docx [file pone.0291343.s008.docx]

Manuscript Title: **Determinants of the Food Insecurity at Household level in Pakistan: A Multilevel Model Approach**

| **#** | **Comments** | **Response** | **Page #** |
| --- | --- | --- | --- |
| **Journal requirement** | | | |
|  | Journal Requirements:  Please review your reference list to ensure that it is complete and correct. If you have cited papers that have been retracted, please include the rationale for doing so in the manuscript text, or remove these references and replace them with relevant current references. Any changes to the reference list should be mentioned in the rebuttal letter that accompanies your revised manuscript. If you need to cite a retracted article, indicate the article’s retracted status in the References list and also include a citation and full reference for the retraction notice. | Checked and corrected |  |
| **Reviewer 2** | | | |
| 1. | Line 99 - Kindly clarify why the questions were asked by the head of the household? it also contradicts with the statement that follows. It reads as if the head of the household asked and responded to the questions, which does not sound right. | The Data regarding the subject matter were taken from the Pakistan Panel Household Survey (PPHS) dataset, which was published. It is not contradicted with statement as the household head has all the information about the member of the house. The result was authenticated, validated, and widely accepted by many research studies.  The datasets used is publicly available. <http://www.pidelms.com/pphs/applyfordata.php> |  |
|  | Is the manuscript presented in an intelligible fashion and written in standard English? | This paper was reviewed by the native English speaker |  |
|  | Please use the space provided to explain your answers to the questions above. You may also include additional comments for the author, including concerns about dual publication, research ethics, or publication ethics. (Please upload your review as an attachment if it exceeds 20,000 characters) | Responded |  |
| **Reviewer 1** | | | |
| 3 | I appreciate the author for the incorporation of all the comments and the revised version. | Many thanks for consideration of our important research work for publication in PloS One Journal. | |

Authors are grateful to the reviewers for their enlightening comments on our previous manuscript submitted to PloS One. We have thoroughly revised the manuscript in light of the comments of the reviewers. Reviewer’s comments and author’s response is provided below;
