# Supplementary material for: Determinants of the food insecurity at household level in Pakistan: A multilevel model approach
Source: PLoS One. 2023 Oct 5;18(10):e0291343. doi: 10.1371/journal.pone.0291343 (PMC10553256; doi:10.1371/journal.pone.0291343)
Supplement: Attachment — Submitted filename: Response to reviwers.docx [file pone.0291343.s009.docx]

Manuscript Title: **Determinants of the Food Insecurity at Household level in Pakistan: A Multilevel Model Approach**

| **#** | **Comments** | **Response** | **Page #** |
| --- | --- | --- | --- |
| **Journal requirement** | | | |
|  | Journal Requirements:  Please review your reference list to ensure that it is complete and correct. If you have cited papers that have been retracted, please include the rationale for doing so in the manuscript text, or remove these references and replace them with relevant current references. Any changes to the reference list should be mentioned in the rebuttal letter that accompanies your revised manuscript. If you need to cite a retracted article, indicate the article’s retracted status in the References list and also include a citation and full reference for the retraction notice. | Checked and corrected | 14-19 |
| **Reviewer 2** | | | |
| 1. | I suggest that line 98 be rephrased as follows: The outcome variables were constructed from the FIES food security module, and the head of the household was the respondent for this study | Thanks for the guidance. We have revised as per the suggestions. | 5 |
|  | 5. Is the manuscript presented in an intelligible fashion and written in standard English?  Reviewer #2: No | We have revised and corrected accordingly. | Whole paper |

Authors are grateful to the reviewers for their enlightening comments on our previous manuscript submitted to PloS One. We have thoroughly revised the manuscript in light of the comments of the reviewers. Reviewer’s comments and author’s response is provided below;
